# A New Degree Needed

**Published:** 1902-08

**Authors:** 


### "A NEW DEGREE NEEDED.

"What is needed is the degree of doctor of achievement, not exactly a mantle of charity, but a hood and gown that can be put upon men who do things, achieve distinction and large results in lines of effort other than philosophy, theology, law, music, literature, and the humanities. These established doctorates have been stretched to meet the desire to honor men whose remote relationship to them compelled the straining. The demand is for a degree to reach out beyond the old scholastic conceptions of the realm of learning into the new world of education that comes through experience and accomplishment, a learning that is real, and that is both helpful and substantial. The colleges have long exhibited their disposition to ally learning and progress, to put the evidence of their recognition upon those who achieve results. Let it then be done fittingly, with more of consonance than is sometimes possible under the limitations of the situation as it exists.

"Finally, let it be kept in mind that the doctorate of achievement would cover the accumulative accomplishment, and so provide a place for benefactors upon whom other degrees would rest with humiliating awkwardness. Here is not the least of the arguments in its favor, let it be said, when every college in the land confesses that its greatest need is money."—*Springfield Republican.*

The foregoing, sent by a friend, and taken from a Massachusetts journal, is so suggestive that it is given a prominent place in this number. It also enables the writer to correct some misapprehension in the minds of several correspondents in relation to the article in the July number in which the conferring of honorary degrees, so common at this season of the year, was sharply criticised.

The position of the writer is simply this. He is not opposed to conferring degrees other than professional, but is decidedly in opposition to the use of the older degrees to fit modern conditions and modern progress.

There can be no objection to the conferring a degree that indicates achievement. It is the one thing needed. The degree conferred on Professor Miller expressed this, and it was, therefore, regarded as one worthily bestowed, honoring the giver and receiver. To confer the degree of LL.D. upon a man simply because he has donated millions to a university, or has done something directly or indirectly in the interest of universities and colleges, is, in effect, to lower the value of all degrees and to make those of an honorary character a byword and a reproach.

When a man or woman has done something that adds to the world's knowledge or its comfort, there should be a degree made to fit the work performed. Let the old degrees stand, for they have their place and the world cannot well dispense with them, but let them be lifted from the mire with which they are fast becoming smirched.

It would be well if the various universities and colleges would take this matter into serious consideration and arrange degrees suitable to all forms of achievement, and then let these be the prizes to be given for an unselfish devotion to works that mean something for the progress of the world.

